# Clinical implication of expression of cyclooxygenase-2 and peroxisome proliferator activated-receptor *γ* in epithelial ovarian tumours

**DOI:** 10.1038/sj.bjc.6602009

**Published:** 2004-07-13

**Authors:** A Sakamoto, Y Yokoyama, M Umemoto, M Futagami, T Sakamoto, X Bing, H Mizunuma

**Affiliations:** 1 Department of Obstetrics and Gynecology, Hirosaki University School of Medicine, 5-Zaifu-cho, Hirosaki, 036-8562, Japan

**Keywords:** COX-2, PPAR*γ*, ovarian tumour, carcinogenesis, 15d-PGJ_2_

## Abstract

Expression of cyclooxygenase (COX)-2 plays a key role in tumorigenesis and development and peroxisome proliferator-activated receptor *γ* (PPAR*γ*) has been implicated in the control of COX-2 expression in some tissues. The aim of this study is to investigate (1) whether expression of COX-2 and PPAR*γ* is associated with ovarian carcinogenesis and progression of ovarian tumours and (2) whether COX-2 expression is controlled through ligand-mediated activation of PPAR*γ* in ovarian carcinoma cells. For this purpose, the presence of COX-2 and PPAR*γ* was immunohistochemically examined in 71 epithelial ovarian carcinomas, 18 borderline tumours and 23 benign tumours and the levels of COX-2 and PPAR*γ* proteins were determined by enzyme immunoassay in four benign tumours, three borderline tumours and 12 carcinomas. The frequency of COX-2 and PPAR*γ* detection was significantly increased and decreased as lesions progressed to carcinoma, respectively. The COX-2 protein was not detected in the three borderline tumours, whereas PPAR*γ* protein was detected in all of them. COX-2 protein was detected in eight of the 12 carcinomas, whereas PPAR*γ* protein was detected in only two cases. In addition, PPAR*γ* protein was not detected in all of the eight carcinomas in which COX-2 protein was detected, suggesting that expression of PPAR*γ* and COX-2 was in a reciprocal relationship. Furthermore, in cultured ovarian carcinoma cells, Western blot revealed that PPAR*γ* and COX-2 expression was regulated conversely as a result of stimulation by 15-deoxy-Δ^12, 14^ PGJ_2_ (15-PGJ_2_), a PPARγ activator. In addition, 15d-PGJ_2_ suppressed tumour necrosis factor-*α*-induced-COX-2 expression, confirming the reciprocal correlation between COX-2 and PPAR*γ*. From these results, it was suggested that PPAR*γ* activation might suppress COX-2 expression via the nuclear factor-*κ*B pathway in the ovarian carcinoma cells and that low expression of PPAR*γ* and high expression of COX-2 might be involved in carcinogenesis and progression of ovarian tumours.

Cyclooxygenase (COX) is a rate-limiting enzyme in prostaglandin (PG) synthesis because of its rapid autoinactivation (Smith and Marnett, 1991). COX has two isoforms, the constitutive COX-1 and the inducible COX-2 (O'Banion *et al*, 1992). COX-1 is expressed in most tissues, whereas COX-2 is largely absent but is responsible primarily for PGs produced in inflammatory sites, suggesting that COX-2 plays a critical role in inflammation (Smith *et al*, 1996). Epidemiological studies have shown 40–50% reduction in mortality from colorectal carcinoma in continuous users of nonsteroid anti-inflammatory drugs (NSAIDs) compared with that of noncontinuous users (Thun *et al*, 1991; Giovannucci *et al*, 1995). Antitumour effect of NSAIDs was caused by inhibition of COX-2 (Taketo, 1998). COX-2 is known to be a promoter of gastrointestinal carcinoma. Many studies suggest that overexpression of COX-2 might be involved in multistep carcinogenesis and tumour progression especially in gastric and colorectal carcinoma (Prescott and White, 1996; van Rees *et al*, 2002). Recently, a progressive development from ovarian serous tumours of low potential malignancy to invasive serous carcinoma has been suggested in parallel to the concept of adenoma–carcinoma sequence in colorectal carcinomas (Hauptmann and Dietel, 2001), although the concept that ovarian carcinogenesis is a linear pathway as for colorectal carcinoma is still contentious. Shigemasa *et al* (2003) reported that COX-2 expression might play an important role in ovarian carcinoma development. Ferrandina *et al* (2002) described that increased COX-2 expression was associated with chemotherapy resistance and outcome in ovarian carcinoma patients and then Denkert *et al* (2002) reported that COX-2 expression was an independent prognostic factor in ovarian carcinoma. More recently, COX-1 has been reported to contribute to carcinoma development in the ovary through stimulation of neovascularisation (Gupta *et al*, 2003).

Peroxisome proliferator-activated receptor *γ* (PPAR*γ*) is a member of a nuclear hormone receptor superfamily that can modulate gene expression upon ligand binding (Subbaramaiah *et al*, 2001). Ligand-mediated activation of PPAR*γ* has been linked to cellular differentiation, apoptosis and anti-inflammatory responses. In colon carcinoma cells, ligand activation of the receptor inhibits cell growth, induces a differentiation response and reverses the malignant phenotype (Sarraf *et al*, 1998). PPAR*γ* agonists and PPAR*γ* overexpression led to a drastic reduction of the cell growth rate in PPAR*γ*-expressing thyroid carcinoma cells (Martelli *et al*, 2002). The nuclear prostanoid 15-deoxy-Δ^12, 14^ PGJ_2_ (15d-PGJ_2_) was identified as a potent natural ligand for the PPAR*γ* (Forman *et al*, 1995; Kliewer *et al*, 1995). 15d-PGJ_2_ was found to induce apoptosis, inhibit proliferation and prevent the growth of human breast carcinoma cells in the nude mouse model (Clay *et al*, 1999). Inhibition of COX-2 and activation of PPAR*γ* inhibited the development of rat mammary gland carcinogenesis (Suh *et al*, 1999; Harris *et al*, 2000). Additionally, independent studies showed that COX-2 and PPAR*γ* are induced and inactivated, respectively, in human breast carcinoma (Jiang *et al*, 2000; Ristimaki *et al*, 2002). Inoue *et al* (2000) proposed that expression of COX-2 was regulated by a negative feedback loop mediated through PPAR*γ* in macrophages. Badawi and Badr (2003) described that the altered expression of COX-2 and PPAR*γ* might influence the development of human breast carcinoma and its progression to metastasis. Recently, PPAR*γ* has been reported to be localised primarily to granulosa cells in ovarian tissue and to be involved in follicular development (Komar *et al*, 2001).

The present study was designed to investigate whether expression of COX-2 and PPAR*γ* is associated with ovarian carcinogenesis and progression of ovarian tumours. We also examined whether COX-2 expression was controlled through ligand-mediated activation of PPAR*γ* in ovarian carcinoma cells. This is the first report describing the relationship between expression of COX-2 and PPAR*γ* in ovarian malignancies.

## MATERIALS AND METHODS

### Study population and tissues

Immunohistochemical examination was performed retrospectively on 112 epithelial ovarian tumours obtained from women who were surgically treated at the Hirosaki University Hospital between 1989 and 2003 after informed consent had been obtained. The tissue specimens included 71 carcinomas, 18 borderline tumours and 23 benign cystadenomas. All patients with epithelial ovarian carcinoma were surgically staged in accordance with the 1988 International Federation of Gynecology and Obstetrics (FIGO) criteria. Patients included in this study had not received any preoperative chemotherapy. The breakdown for stages of ovarian carcinomas consisted of 40 patients with stage I, seven with stage II, 18 with stage III, six with stage IV. Histological types were classified into 30 cases with serous cystadenocarcinoma, 11 with mucinous cystadenocarcinoma, 17 with endometrioid adenocarcinoma, 12 with clear cell adenocarcinoma and one with undifferentiated adenocarcinoma. All patients with ovarian carcinoma received postoperative chemotherapy combining cisplatin (60 mg m^−2^), epirubicin (40 mg m^−2^) and cyclophosphamide (300 mg m^−2^). The duration of follow-up ranged from 8 to 156 months (median, 54 months). The mean age of patients with ovarian carcinoma at surgery was 54.1 years (range, 28–78 years). Of the 18 borderline tumours, four were serous and 14 mucinous. Of the 23 benign cystadenomas, nine were serous and 14 mucinous.

### Immunohistochemical staining of COX-2 and PPAR*γ*

Anti-COX-2 (Immuno-Biological Laboratories, Gunma, Japan) and anti-PPAR*γ* (Cayman Chemical, Ann Arbor, MI, USA) antibodies were used at a concentration of 5 *μ*g ml^−1^, respectively. All samples surgically obtained for immunohistochemistry were immediately fixed in formaldehyde and embedded in paraffin. Sections 6 *μ*m thick were routinely passed through xylene and a graded alcohol series and placed in 0.01 M citrate buffer, pH 6.0, and heated at 500 W in a microwave oven for 5 min to retrieve tissue antigen. The sections were treated with 0.3% hydrogen peroxide (H_2_O_2_) in methanol for 10 min to quench the endogenous peroxidase activity within the tissue. Nonspecific binding sites were blocked with 1% bovine serum albumin (BSA) and 20% heat-inactivated goat serum in phosphate-buffered saline (PBS) for 30 min at room temperature (RT). The sections were then stained for COX-2 or PPAR*γ* by the avidin–biotin–peroxidase complex method using the appropriate antibodies as reported previously (Yokoyama *et al*, 2003). Anti-COX-2 antibody was applied for 12 h at 4°C in a moist chamber. Anti-PPAR*γ* antibody was applied for 1 h at 37°C. The binding sites of peroxidase were visualized with 0.02% diaminobenzidine (DAB) (Sigma-Aldrich, St Louis, MO, USA) as a chromogen in Tris-HCl buffer, pH 7.6 containing 0.03% H_2_O_2_. The sections were then counterstained with haematoxylin for microscopic examination. As negative control, preimmune rabbit serum was used instead of the antibody. As positive control for COX-2 and PPAR*γ* staining, formalin-fixed paraffin-embedded sections of colon carcinoma and urinary bladder carcinoma were stained by the same procedure, respectively. Two observers (AS and YY) independently evaluated and interpreted the results of immunohistochemical staining, without the knowledge of the clinical data of each patient. Cases in which more than 10% of tumour cells were as strongly immunoreactive as positive control cells were considered positive.

### Measurement of levels of COX-2 and PPAR*γ*

Analysis of levels of COX-2 and PPAR*γ* in the ovarian tumours was carried out by enzyme immunoassay (EIA). Four benign tumours, three borderline tumours and 12 carcinomas kept at −80°C were used for this experiment. For measurement of COX-2 and PPAR*γ* levels, the tissue specimens (100 mg) were minced, sonicated in 10 mM Tris-HCl, pH 7.4, 0.5 M NaCl and centrifuged at 10 000 **g** for 15 min at 4°C to separate cell debris and the fat layer. The resulting supernatants were used for analysis. EIA for COX-2 and PPAR*γ* was carried out using a human COX-2 assay kit (Immuno-Biological Laboratories, Gunma, Japan) and a TransAM™ PPAR*γ* Transcription Factor Assay kit (Active Motif, Carlsbad, NM, USA), respectively, according to each manufacturer's instruction. Colour intensity was measured at 450 nm using BIORAD Model 550 microplate reader (Bio-Rad Laboratories, Tokyo, Japan) and the levels of COX-2 and PPAR*γ* were expressed as ng per mg protein and *μ*g per cell extract per well, respectively. The range of measurement sensitivity in COX-2 and PPAR*γ* levels is 2.15 to 275 ng ml^−1^ and 0.75 to 7.5 *μ*g (cell extract)^−1^ (well)^−1^, respectively. Standard curves and positive and negative controls were generated for COX-2 and PPAR*γ* and assayed simultaneously with the samples.

### Cell culture

OVCAR-3 cells were obtained from the American Type Culture Collection and the ovarian carcinoma cell line, which was derived from serous adenocarcinoma and was established at the Hirosaki University Hospital, was used. Cells were seeded at 1.2 × 10^4^ cells cm^−2^ and grown in RPMI 1640 medium supplemented with 10% (v v^−1^) fetal bovine serum (FBS), 100 U ml^−1^ penicillin and 100 mg ml^−1^ streptomycin, at 37°C in a water-saturated atmosphere with 5% CO_2_/95% air.

### 15d-PGJ_2_ treatment

After 24 h, the medium was replaced by the fresh medium (10% FBS) containing 15d-PGJ_2_ (Alexis Biochemicals, San Diego, CA, USA) at a final concentration of 0.1, 1, 10 or 20 *μ*M. Cells were exposed to 15d-PGJ_2_ at the indicated concentrations for 72 h of treatment. As a negative control, cells were cultured in a medium without 15d-PGJ_2_. For each cell culture set, expression of COX-2 and PPAR*γ* in 15d-PGJ_2_-treated and untreated cells was examined using Western blot analysis.

### Tumour necrosis factor-*α* and 15d-PGJ_2_ treatment

The medium was replaced with serum-free medium 12 h before stimulation. OVCAR-3 cells were then treated with 20 ng ml^−1^ tumour necrosis factor (TNF)-*α* (Strathmann Biotec AG, Hamburg, Germany) in the presence or absence of 20 *μ*M 15d-PGJ_2_ for 24 h. As a negative control, cells were cultured in a medium without TNF-*α* and 15d-PGJ_2_. For each cell culture set, expression of COX-2 was examined using Western blot analysis.

### Western blot analysis

For Western blot analysis, cells were washed with PBS after the incubation period, scraped into 10 mM Tris-HCl, pH 7.4, 0.5 M NaCl, and sonicated three times for 30 s using Ultra S Homogenizer model (Taitec, Nagoya, Japan). After centrifugation at 10 000 **g** for 15 min at 4°C, the supernatants were used for protein assay. The protein concentration was determined using Bradford method. The protein samples (50 *μ*g) were run through 12.5% sodium dodecyl sulphate (SDS)–polyacrylamide gel electrophoresis. After electrophoretic transfer of the protein to nitrocellulose membrane, nonspecific binding was blocked by incubation with 3% gelatin in 20 mM Tris-HCl, pH 7.5, 0.5 M NaCl (TBS) for 1 h at RT. After being washed three times with TBS containing 0.05% Tween 20 (TTBS), the blots were probed with a rabbit polyclonal IgG specific for human COX-2 (Immuno-Biological Laboratories, Gumma, Japan) or a PPAR*γ* polyclonal antibody (Cayman Chemical, Ann Arbor, MI, USA) for 2 h. The blots were also probed with a monoclonal anti-*β*-actin antibody (SIGMA, St Louis, MO, USA) to be relatively quantified. *β*-Actin was used as a loading control. The membranes were then washed three times with TTBS and incubated for 1 h at RT with a biotinylated anti-rabbit immunogloblin (Vector Laboratories, Burlingame, CA, USA) for COX-2 and PPAR*γ* and a biotinylated anti-mouse immunogloblin (Vector Laboratories, Burlingame, CA, USA) for (*β*-actin, respectively. After being washed three times with TTBS, the membrane were transferred to VECTERSTA1N ABC Reagent (Vector Laboratories, Burlingame, CA, USA) and incubated in this solution for 30 min at RT. Diaminobenzidine was used as a substrate of peroxidase.

### Statistical analysis

Statistical analysis was carried out by *χ*^2^-test or Fisher's exact probability test. A result was deemed significant at *P*<0.05.

## RESULTS

### Detection of COX-2 and PPAR*γ* in benign, borderline tumours and carcinoma

**Figure 1:**
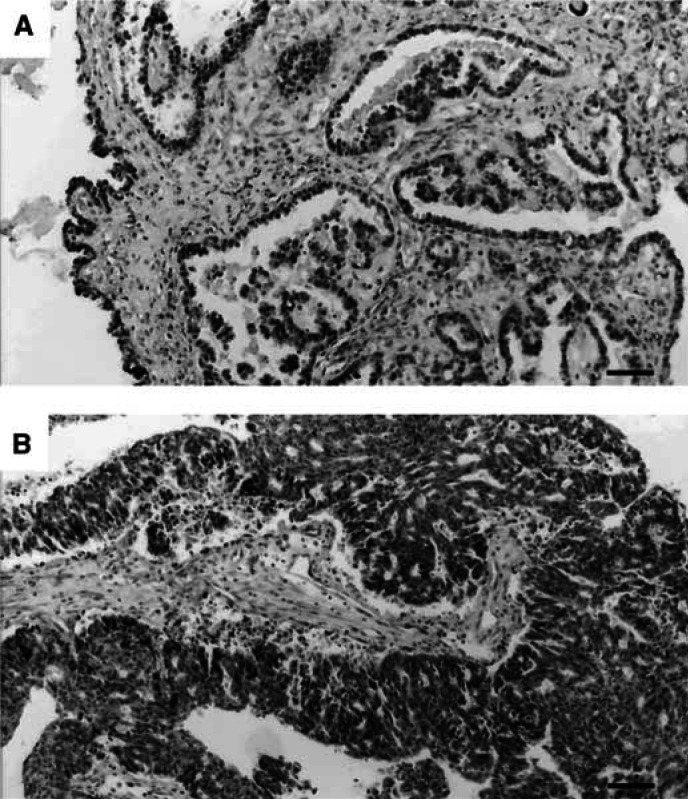
Immunohistochemical staining of COX-2 and PPAR*γ* in ovarian carcinoma. Positive staining of COX-2 (**A**) and PPAR*γ* (**B**) in carcinoma tissues (scale bar, 50 *μ*m). They are representative of all the positive samples. Tissues demonstrated in (**A**) and (**B**) are a serous adenocarcinoma.

COX-2 and PPAR*γ* were homogeneously stained in the cytoplasm of tumour cells in positive cases (Figure 1

). The frequencies of COX-2 and PPAR*γ* detection in ovarian tumours are demonstrated in Table 1

**Table 1:** Detection of COX-2 and PPAR*γ* in ovarian tumours

**Tissue**	**No. of cases**	**Case no. (%) of COX-2 positive**	**Case no. (%) of PPAR*γ* positive**
Benign tumour	23	3 (13.0)	1 (4.5)
Borderline tumour	18	3 (16.7)	14 (77.8)
Carcinoma	71	28 (39.4)	31 (43.7)

The incidence of COX-2 detected in carcinomas was significantly higher than that detected in benign tumours (*P*<0.02) and higher with a marginal significance than that detected in borderline tumours (*P*=0.058). The frequency of PPAR*γ* detected in borderline tumours was significantly higher than that detected in benign tumours and carcinomas, respectively (*P*<0.0001, *P*<0.01, respectively).

. There was no significant difference in COX-2 positivity between samples of benign and borderline tumours (Table 1). The incidence of COX-2 detected in carcinomas was significantly higher than that detected in benign tumours (Table 1, *P*<0.02) and higher with a marginal significance than that detected in borderline tumours (Table 1, *P*=0.058). On the other hand, the frequency of PPAR*γ* detected in borderline tumours was significantly higher than that detected in benign tumours and carcinomas, respectively (Table 1, *P*<0.0001, *P*<0.01, respectively).

COX-2 and PPAR*γ* positivity in carcinomas was not correlated with clinical factors such as stage, histological type, lymph node metastasis and recurrence (Table 2

**Table 2:** Correlation between expression of COX-2 and PPAR*γ* and clinicopathological factors in ovarian carcinoma

**Factor**	**No. of cases**	**Case no. (%) of COX-2 positive**	**Case no. (%) of PPAR*γ* positive**
*Stage*			
I/II	47	20 (42.6)	21 (44.7)
III/IV	24	8 (33.3)	9 (37.5)
*Histological type*			
Serous	30	14 (46.7)	10 (33.3)
Mucinous	11	3 (27.3)	6 (54.5)
Endometrioid	17	6 (353)	10 (58.8)
Clear cell	12	5 (41.7)	4 (33.3)
Undifferentiated	1	0	0
*Lymph node metastasis*			
Positive	10	6 (60.0)	4 (40.0)
Negative	61	22 (36.1)	26 (42.6)
*Outcome*			
Recurrent	19	6 (41.6)	8 (42.1)
Nonrecurrent	52	22 (42.3)	22 (42.3)

COX-2 and PPAR*γ* positivity in carcinomas was not correlated with clinical factors such as stage, histological type, lymph node metastasis and recurrence.

).

### Determination of COX-2 and PPAR*γ* protein levels in ovarian tumour tissues

Levels of COX-2 and PPAR*γ* proteins were determined by EIA in four benign tumours, three borderline tumours and 12 carcinomas. COX-2 and PPAR*γ* proteins were not detected in the benign tumours at all (Table 3

**Table 3:** Determination of COX-2 and PPAR*γ* proteins in ovarian tumours

**Tissue**	**Case**	**Amount of COX-2 protein (ng per mg protein)**	**Amount of PPAR*γ* protein (*μ*g (cell extract)^−1^ (well)^−1^)**
Benign cystadenoma	1	ND	ND
	2	ND	ND
	3	ND	ND
	4	ND	ND
			
Borderline tumour	1	ND	92.9
	2	ND	74.7
	3	ND	57.9
			
Carcinoma	1	3.91	ND
	2	3.69	ND
	3	2.65	ND
	4	2.32	ND
	5	2.22	ND
	6	1.98	ND
	7	1.72	ND
	8	1.25	ND
	9	ND	ND
	10	ND	ND
	11	ND	70.1
	12	ND	60.2

ND stands for not detectable.

). The COX-2 protein levels of the three borderline tumours were below the measurement sensitivity, whereas PPAR*γ* protein was detected in all of those ones (Table 3). COX-2 protein was detected in eight of the 12 carcinomas and the remaining four carcinomas lacked COX-2 protein (Table 3). In contrast, 10 of the 12 carcinomas lacked PPAR*γ* protein and PPAR*γ* protein was detected in only two carcinomas (Table 3). The level of PPAR*γ* protein was below the measurement sensitivity in all of the eight carcinomas in which COX-2 protein was detected, whereas COX-2 protein lacked in the two carcinomas in which PPAR*γ* protein was detected (Table 3).

### Effect of 15d-PGJ_2_ on COX-2 and PPAR*γ* expression in cultured ovarian carcinoma cells

**Figure 2:**
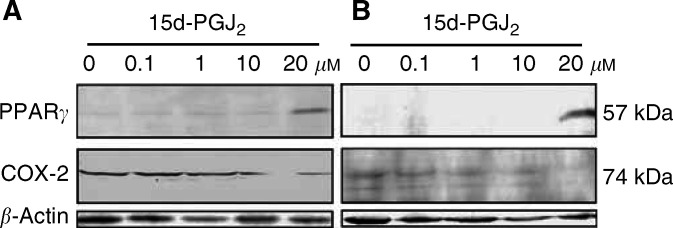
Effect of 15d-PGJ_2_ on COX-2 and PPAR*γ* expression in OVCAR-3 (**A**) and the ovarian carcinoma cell line established at our institute (**B**). In both cell lines, PPAR*γ* expression was increased according to 15d-PGJ_2_ concentrations, whereas COX-2 expression was decreased according to 15d-PGJ_2_ concentrations. Results shown are representative of two separate experiments with two cell lines, *β*-Actin was used as a loading control.

To examine whether COX-2 expression is controlled through PPAR*γ* activation in ovarian carcinoma cells, OVCAR-3 cells and the ovarian carcinoma cells established at our hospital were cultured in a semiconfluent state in normal culture medium or in media supplemented with 0.1–20 *μ*M 15d-PGJ_2_, a PPAR*γ* activator as described in Materials and Methods. Western blot analysis revealed that expression of PPAR*γ* and COX-2 was increased and decreased, respectively according to 15d-PGJ_2_ concentrations (Figure 2

), suggesting that 15d-PGJ_2_ activated PPAR*γ* in both cell lines and PPAR*γ* activation might result in suppression of COX-2 expression in the cells. Results shown in Figure 2 are representative of two separate experiments with two cell lines. *β*-Actin was used as a loading control.

### Suppressive effect of 15d-PGJ_2_ on cytokine-induced COX-2 expression in cultured ovarian carcinoma cells

**Figure 3:**
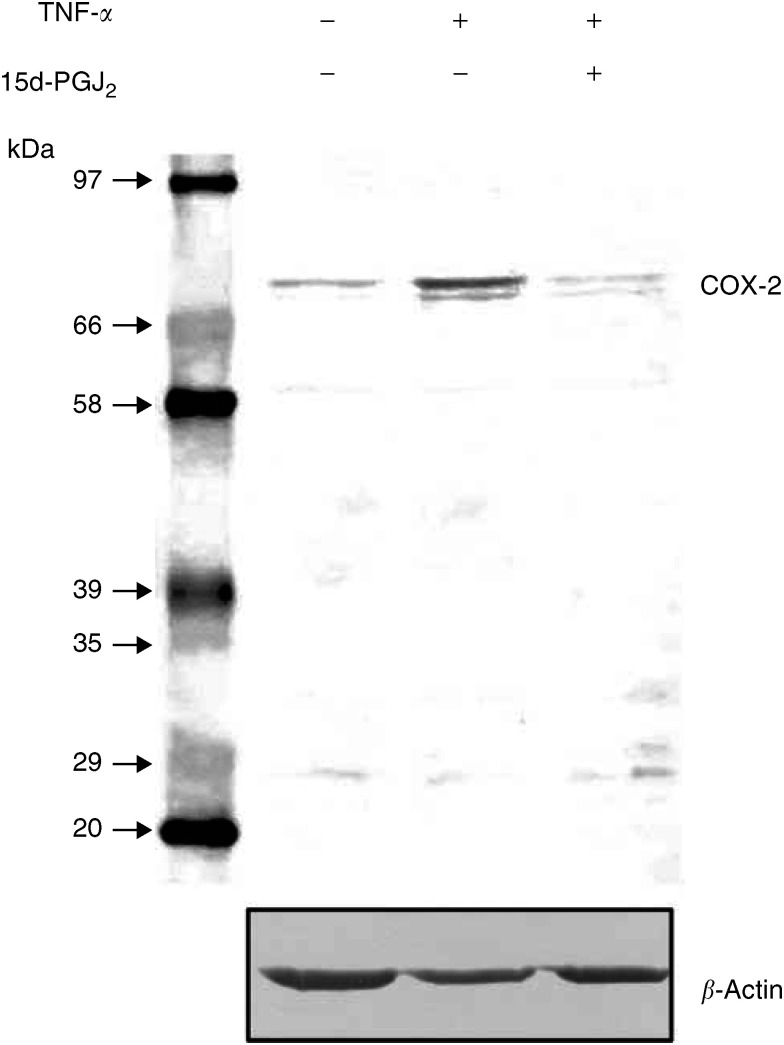
Suppressive effect of 15d-PGJ_2_ on TNF-*α*-induced COX-2 expression in OVCAR-3 cells. TNF-*α* induced COX-2 expression in OVCAR-3 cells. Addition of 20 *μ*M 15d-PGJ_2_ to the media supplemented with TNF-*α* suppressed TNF-*α*-induced-COX-2 expression. *β*-Actin was used as a loading control.

TNF-*α*, an inflammatory cytokine, is known to increase COX-2 expression via nuclear factor NF*κ*B pathway. To examine whether NF*κ*B pathway is related to the signalling between PPAR*γ* activation and COX-2 expression, OVCAR-3 cells were cultured in a semiconfluent state in normal culture medium or in media supplemented with TNF-*α* in the presence or absence of 20 *μ*m 15d-PGJ_2_ as described in Materials and Methods. Tumour necrosis factor-alpha induced COX-2 expression in OVCAR-3 cells (Figure 3

). Addition of 15d-PGJ_2_ to the media supplemented with TNF-*α* suppressed TNF-*α*-induced-COX-2 expression (Figure 3). *β*-Actin was used as a loading control.

## DISCUSSION

The present study clearly demonstrated that the frequency and the degree of PPAR*γ* expression were significantly decreased as lesions progressed from a borderline tumour to carcinoma, and those of COX-2 expression were significantly increased as lesions progressed from a benign tumour to carcinoma, suggesting that low expression of PPAR*γ* and high expression of COX-2 in precancerous lesions might be involved in progression of ovarian tumours to carcinoma. Independent studies have shown that induction of COX-2 and inactivation of PPAR*γ* occurred during the development and progression of human breast carcinoma (Jiang *et al*, 2000; Ristimaki *et al*, 2002). On the other hand, inhibition of COX-2 and activation of PPAR*γ* has been shown to prevent mammary carcinogenesis in experimental animals (Suh *et al*, 1999; Harris *et al*, 2000). Additionally, COX-2 selective inhibitors and PPAR*γ* ligands could significantly attenuate the growth of human breast carcinoma cells (Elstner *et al*, 1998; Howe *et al*, 2001). Thus it has been suggested that high expression of PPAR*γ* and low expression of COX-2 in the tumours might be involved in attenuating the capacity of the tumours to develop more malignant nature. Badawi and Badr (2003) described that an increase of COX-2 expression and a decrease of PPAR*γ* expression in breast carcinoma tissues were paralleled by increases in the tissue levels of PGE_2_ and decreases in 15d-PGJ_2_ and their altered expressions might influence the development of human breast carcinoma.

COX-2 overexpression has been observed in many tumour types including gynaecological malignancies. Mechanistic study suggested that expression of COX-2 in uterine cervical carcinoma cells downregulated apoptotic processes and thus enhanced tumour invasion and metastasis (Affney *et al*, 2001). Li *et al* (2004) described that while COX-2 protein was not detected in normal epithelium of the ovary, its protein was frequently expressed in ovarian epithelial carcinoma, suggesting that it might contribute to the carcinoma development or progression. Matsumoto *et al* (2001) found a significant correlation between expression of vascular endothelial growth factor (VEGF) and COX-2 in ovarian neoplasms and suggested that an increased expression of COX-2 might be associated with malignant transformation and tumorigenesis through the activation of VEGF. Denkert *et al* (2002) reported that expression of COX-2 was immunohistochemically detected in 42% of ovarian carcinomas and in 37% of borderline tumors, and described that COX-2 expression was an independent prognostic factor in human ovarian carcinoma. Although we found high frequency of COX-2 expression in ovarian carcinoma, we did not show the close relationship between expression of COX-2 and metastasis or recurrence in ovarian carcinoma. Large-scale prospective and retrospective studies are needed to clarify whether COX-2 expression is of practical utility as a prognostic factor.

PPAR*γ* plays a crucial role in apoptosis and differentiation of a variety of cells. Induction of differentiation has been observed in several malignant cells as a result of stimulation by PPAR*γ* (Mueller *et al*, 1998). PPAR*γ* activation resulted in apoptosis of choriocarcinoma cells (Keelan *et al*, 1999), prostate carcinoma cells (Kubota *et al*, 1998), leukaemic cells (Asou *et al*, 1999) and gastric carcinoma (Sato *et al*, 2000). More recently, Martelli *et al* (2002) reported that arrest of G1 cell cycle was observed and apoptosis was induced in thyroid carcinoma cells transfected with PPAR*γ*. Breast carcinoma cells treated with PPAR*γ* agonist show dramatic morphological changes, and express E-cadherin and b-casein, markers of breast cell differentiation (Elstner *et al*, 1998; Clay *et al*, 1999). Furthermore, it has been reported that differentiation and reversal of malignant changes were induced in CX-1 colonic tumour cells treated with PPAR*γ* agonist in Swiss nude mice (Sarraf *et al*, 1998). Zander *et al* (2002) described that activation of PPAR*γ* transiently induced N-cadherin, the glioma differentiation marker, in human and rat glioma cells and in parallel, a subset of surviving cells showed *de novo* outgrowth of processes resembling astrocyte-like morphology based on PPAR*γ* activation. Taking these results together including ours, activation of PPAR*γ* and inhibition of COX-2 may be favourable for guiding neoplastic cells towards redifferentiation.

PPAR*γ* signalling has been implicated in the control of COX-2 expression in certain tissues, although the exact mechanism that underlies PPAR*γ* regulation of COX-2 expression remains to be elucidated. To date, there are several reports suggesting a reciprocal interaction between COX-2 expression and PPAR*γ* activity (Inoue *et al*, 2000; Ikawa *et al*, 2001; Yang and Frucht, 2001). However, it is still unclear whether COX-2 expression is controlled through PPAR*γ* signalling in ovarian carcinoma cells. We investigated whether PPAR*γ* activity was involved in COX-2 regulation in human ovarian carcinoma cells, and found that 15d-PGJ_2_, a PPAR*γ* ligand, reduced COX-2 expression in a dose-related manner, suggesting that COX-2 expression was regulated through PPAR*γ* activity in ovarian carcinoma cells. The inhibitory effect of PPAR*γ* activation on COX-2 expression is also reported in HT-29 human colon carcinoma cells (Yang and Frucht, 2001). Furthermore, it has been shown that NSAID suppress IL-1*β*-induced COX-2 expression (Xu *et al*, 1999), and inhibits TNF-*α*-induced COX-2 expression (Paik *et al*, 2000), suggesting that expression of COX-2 is under the control mechanisms by both cytokines and PPAR*γ* systems. In fact, Inoue *et al* (2000) demonstrated that 15d-PGJ_2_ suppressed COX-2 promoter activity by interfering with the NF*κ*B signalling pathway, and that transfection of a PPAR*γ* expression vector into the endothelial cells resumes the suppressive regulation of *COX-2* gene by 15d-PGJ_2_. Tumour necrosis factor-alpha is a strong inducer of COX-2 expression by stimulating the NF*κ*B system (Yamamoto *et al*, 1995). As shown in Figure 3, the present result showed that COX-2 was induced by TNF-*α* in ovarian carcinoma cells and that TNF-*α*-induced COX-2 expression was suppressed by 15d-PGJ_2_, suggesting that COX-2 expression was under the control of PPAR*γ* and NF*κ*B pathway in the ovarian carcinoma.

In conclusion, the reciprocal correlation between COX-2 and PPAR*γ* found in the present study implicates that COX-2 and PPAR*γ* may contribute to ovarian carcinoma induction. Thus, the present results suggested that ligand-mediated PPAR*γ* activation suppressed COX-2 expression via the NF*κ*B pathway in the ovarian carcinoma cells, and that high expression of PPAR*γ* and low expression of COX-2 might play an important role in inhibiting ovarian carcinogenesis.
